# Genetic Characterization of Avian Influenza A(H5N6) Virus Clade 2.3.4.4, Russia, 2018

**DOI:** 10.3201/eid2512.190504

**Published:** 2019-12

**Authors:** Ivan M. Susloparov, Natalia Goncharova, Natalia Kolosova, Alexey Danilenko, Vasiliy Marchenko, Galina Onkhonova, Vasiliy Evseenko, Elena Gavrilova, Rinat A. Maksutov, Alexander Ryzhikov

**Affiliations:** State Research Center of Virology and Biotechnology Vector, Koltsovo, Russia

**Keywords:** avian influenza virus, highly pathogenic, HPAIV, H5N6, wild birds, surveillance, clade 2.3.4.4, resassortment, phylogenetic analysis, viruses, Russia, respiratory infections, zoonoses, influenza

## Abstract

Timely identification of pandemic influenza threats depends on monitoring for highly pathogenic avian influenza viruses. We isolated highly pathogenic avian influenza A(H5N6) virus clade 2.3.4.4, genotype G1.1, in samples from a bird in southwest Russia. The virus has high homology to human H5N6 influenza strains isolated from southeast China.

Highly pathogenic avian influenza (HPAI) H5 virus continues to evolve and pose a threat to animals and humans. Since 2008, HPAI H5 viruses of clade 2.3.4.4 with various neuraminidase (NA) subtypes have become widespread throughout the world and have caused mass epizootics, including in Russia, where these viruses have been reported since 2014 (*1*). In 2013, H5N6 virus began circulating in China (*2*), and a case of human disease was recorded there in 2014. Since then, 23 cases of H5N6 infection in humans, including 7 fatalities, have been confirmed in China (*3*).

In October 2018, we collected cloacal swab samples from aquatic birds around the Volga River Basin in the Saratov region of Russia (51°26′11.7″N, 46°06′49.9″E). We isolated avian H5 influenza virus from 1 sample from a common gull (*Larus canus*) by using embryonic chicken eggs. We used whole-genome sequencing to extract the virus DNA and conducted a phylogenetic analysis against strains available in the GISAID EpiFlu database (http://www.gisaid.org). We submitted genetic data on the virus, A/common gull/Saratov/1676/2018, to the GISAID EpiFlu database (identification no. EPIISL336925).

Using H5 clade nomenclature designated by the World Health Organization/World Organisation for Animal Health/Food and Agriculture Organization H5 Evolution Working Group (*4*), our phylogenetic analysis showed that hemagglutinin (HA) gene of A/common gull/Saratov/1676/2018 clusters with HPAI viruses in clade 2.3.4.4 H5N6-H5/Major lineage. Our analyses also show this strain belongs to a new HA subgroup that includes human H5N6 viruses isolated in Guangxi and Guangdong Provinces, China, in 2018 (Appendix Figure 1, Table 1). This subgroup is not represented by existing candidate vaccine viruses (CVVs) (*5*,*6*).

The NA gene of A/common gull/Saratov/1676/2018 appears to originate from H6N6 viruses circulating in Asia during 2010–2011 (Appendix Figure 2) and contains the deletion from positions 59–69 in the stalk region. The polymerase basic (PB) 2 gene segment also appears to have originated from an H6 subtype (Appendix Figure 3). The internal gene segments PB1, polymerase (PA), nucleoprotein (NP), matrix (M), and nonstructural protein (NSP) appear to have evolved from HPAI H5 virus clade 2.3.2.1 (Appendix Figures 4–8). The 8-segment constellation leads us to classify this strain into a G1.1 genotype, as described by Bi et al. (*6*).

We conducted a comparative genomic analysis of A/common gull/Saratov/1676/2018 against H5N6 CVVs; the most pronounced differences were several amino acid substitutions associated with potential changes in antigenic properties. We also detected unique mutations in HA D54N, L115Q, L/Q138T, P141A, N183S, and N189D, including a combination of S121Y and I151T. We noted other mutations, including HA L129S, K/M/T140V (H5 numbering), and NA N86K (N6 numbering), which could be associated with antigenic drift. 

A/common gull/Saratov/1676/2018 had an HA polybasic proteolytic cleavage site, PLRERRRKR/G, and showed highly pathogenic properties by killing chicken embryos within 48 hours. We also identified amino acid changes associated with increased virulence to mammals (*7*,*8*), including 9 mutations in the PB2 gene, 8 in the PB1 gene, 7 in the NSP gene, 3 in the M gene, 2 in the PA gene, 1 in the HA gene, and 1 in the NA gene, along with the 59–69 deletion, an 80–84 deletion in NS1, and an NS1 ESEV terminal motif. These changes also appear in most H5N6 CVVs (Appendix Table 2).

Comparative analysis of A/common gull/Saratov/1676/2018 against H5N6 CVVs revealed similarity in the presence of genetic elements associated with receptor binding properties. A/common gull/Saratov/1676/2018 and most CVVs had the motif QS(R)G at the receptor-binding site (nt 222–224), which is associated with an avian-like α2,3-SA receptor-binding preference (*6*). The amino acid changes in D94N, S133A, and T156A in the HA of A/common gull/Saratov/1676/2018 and most H5N6 CVVs are associated with increased binding of the virus to human-like α2,6-SA receptors (*7*). Our analysis suggests that A/common gull/Saratov/1676/2018 retains its avian status but has several mutations that potentially increase its affinity for α2,6-SA, which could indicate an affinity for both avian- and human-type receptors. 

We evaluated the phenotypic properties of the virions by kinetics measurement with surface plasmon resonance to assess their ability to bind to receptor analogs α2,3-SA and α2,6-SA (*9*). The equilibrium dissociation constant for 3′-Sialyl-N-acetyllactosamine is 12.2 (SD ± 0.7 nmol/L) and for 6′-Sialyl-N-acetyllactosamine is 43.3 (SD ± 2.8 nmol/L) (Appendix). These values show that A/common gull/Saratov/1676/2018 has prevalent affinity for the avian-like receptor with lower, but increased, affinity for the human-like receptor, compared with H5N1 strain A/rook/Chany/32/2015 clade 2.3.2.1.C.

Analysis of homology of A/common gull/Saratov/1676/2018 with H5N6 strains available from GISAID showed that all 8 gene segments clustered with human H5N6 strains isolated in southeast China in 2018. We noted 99% homology with human strain A/Guangxi/32797/2018 for all genes, a genetic similarity that raises the question of which pathway led to the spread of the virus. We believe A/common gull/Saratov/1676/2018 was transferred to eastern Russia through northeast Siberia, where HPAI H5N8 clade 2.3.4.4.A was detected in 2018 (*10*), the same pathway through which H5N8 virus was transferred from Southeast Asia to Europe. These viral pathogens could be spread by migratory birds over long distances along flyways from southern China to southwestern Russia during a migration season. Our study indicates that emerging H5N6 viruses are a potential threat to public health.

Appendix Additional information on genetic characterization of avian influenza A(H5N6) virus clade 2.3.4.4, Russia, 2018.
