# Supplementary material for: Genetic Characterization of Avian Influenza A(H5N6) Virus Clade 2.3.4.4, Russia, 2018
Source: Emerg Infect Dis. 2019 Dec;25(12):2338–9. doi: 10.3201/eid2512.190504 (PMC6874274; doi:10.3201/eid2512.190504)
Supplement: Appendix — Additional information on genetic characterization of avian influenza A(H5N6) virus clade 2.3.4.4, Russia, 2018. [file 19-0504-Techapp-s1.pdf]

Article DOI: https://doi.org/10.3201/eid2512.190504

Genetic Characterization of Avian Influenza A(H5N6) Virus Clade 2.3.4.4, Russia, 2018

Appendix

Measurement of Equilibrium Dissociation Constants

To determine receptor preference, we measured binding kinetics of virions to receptor analogs by surface plasma resonance on a ProteOn XPR36 (Bio-Rad, https://www.bio-rad.com) with a NeutrAvidin chip (Bio-Rad) and 3′-Sialyl-N-acetyllactosamine and 6′-Sialyl-N-acetyllactosamine biotinylated receptor analogs (Dextra, https://www.dextrauk.com). We immobilized α2–3 and α2–6 glycans on the NLC chip in sodium phosphate buffer (pH 7.4) at a concentration of 100 µg/mL. We injected 5 dilutions of purified virus sample in the same buffer at a flow rate of 70 µL/min with 350 seconds contact time for association. Dissociation lasted 600 seconds at the same flow rate. We added oseltamivir (20 nmol/L) to inhibit neuraminidase. We analyzed data with the ProteOn Manager (Bio-Rad) software using Langmuir kinetics calculations model (Appendix Figure 9). We calculated equilibrium dissociation constants (K_D) as ratio of dissociation and association constants: $K_D = k_d/k_a$. We used 3 independent surface plasma resonance runs to verify the equilibrium dissociation constants.

K_D for 3′SLN and 6′SLN of A/common gull/Saratov/1676/2018

K_D for 3′SLN = 12.2 ± 0.7 nmol/L

K_D for 6′SLN = 43.3 ± 2.8 nmol/L

K_D for 3′SLN and 6′SLN of A/rook/Chany/32/2015

K_D for 3′SLN = 0.2 ± 0.02 µmol/L

K_D for 6′SLN = 6.3 ± 0.1 µmol/L

The data confirms preferential binding of both strains to α2,3-SA.

Appendix Table 1. Comparison of gene segments of avian influenza A(H5N6) virus clade 2.3.4.4 isolated in Russia, 2018 with human influenza A H5N6 viruses*

Gene segment	GISAID no.	Guangxi/32797/ 2018	Guangxi/31906/ 2018	Guangdong/ 18SF020/2018	Guangxi/13486/2017	Jiangsu/32888/2018
HA	EPI1355418	1,763/1,773 (99)	1,753/1,772 (98)	1,753/1,774 (98)	1,744/1,774 (98)	1,723/1,775 (97)
NA	EPI1355420	1,430/1,432 (99)	1,416/1,432 (98)	1,411/1,432 (98)	1,418/1,432 (99)	1,378/1,435 (96)
PB2	EPI1355415	2,330/2,335 (99)	2,324/2,341 (99)	2,303/2,326 (99)	2,303/2,326 (99)	2,283/2,326 (98)
PB1	EPI1355416	2,290/2,301 (99)	2,310/2,341 (98)	2,237/2,274 (98)	2,256/2,274 (99)	2,300/2,331 (98)
PA	EPI1355417	2,225/2,233 (99)	2,209/2,233 (98)	2,123/2,151 (98)	2,132/2,151 (99)	2,017/2,214 (91)
NP	EPI1355419	1,540/1,543 (99)	1,548/1,565 (98)	1,550/1,565 (99)	1,551/1,565 (99)	1,550/1,565 (99)
M	EPI1355421	1,022/1,027 (99)	1,018/1,027 (99)	1,024/1,028 (99)	1,003/1,012 (99)	1,018/1,027 (99)
NSP	EPI1355422	868/870 (99)	865/875 (98)	870/875 (99)	872/875 (99)	871/876 (99)

*Avian influenza A(H5N6) isolated in this study, A/common gull/Saratov/1676/2018 in Global Initiative on Sharing All Influenza Data database. Values for nucleic sequence homology of each gene segment expressed as gene segments of A/common gull/Saratov/1676/2018 versus gene segments of human A(H5N6). Values in parentheses represent % identity. GSAID, Global Initiative on Sharing All Influenza Data HA, hemagglutinin; M, matrix; NA, neuraminidase; NP, nucleoprotein; NSP, nonstructural protein; PA, polymerase; PB1, polymerase basic 1; PB2, polymerase basic 2.

Appendix Table 2. Amino acid changes in proteins of avian influenza A(H5N6) compared with the closest homologue and H5N6 candidate vaccine viruses*

	Human H5N6 virus strains				Avian H5N6 virus strains			
Gene	A/Hubei/ 29578/ 2016†	A/Fujian-Sanyuan/ 21099/ 2017†	A/Sichuan/ 26221/ 2014†	A/Guangxi/ 32797/ 2018†	A/chicken/ Vietnam/ NCVD-15A59/2015†	A/duck/ Hyogo/ 1/2016†	A/common gull/ Saratov/ 1676/2018	Phenotypic characteristics
HA (H5 no.)								
D54N	D	D	D	N	D	D	N	Creates a potential N-glycosylation site
D94N	N	S	N	N	N	N	N	Increased virus binding to α2–6
L115Q	L	L	L	Q	L	L	Q	Antigenic drift
S121Y	S	S	S	S	S	S	Y	Together with I151T antigenic drift
S123P	P	P	T	S	P	P	S	Increased virus binding to α2–6
126 Del	Del	E	E	Del	E	E	Del	Creates a potential N-glycosylation site
L129S	S	L	L	S	L	Del	S	Position associated with antigenic drift
S133A	A	A	A	A	A	A	A	Increased virus binding to α2–6
L/Q138T	L	Q	Q	T	Q	Q	T	Position associated with antigenic drift
K/M/T140V	K	T	T	V	M	V	V	Position associated with antigenic drift
P141A	P	P	P	A	P	P	A	Antigenic drift
I151T	T	I	I	T	T	T	T	With S121Y, antigenic drift; with 129Del, host specificity shift
T156A	A	A	A	A	A	A	A	Increased virus binding to α2–6
N183S	N	N	N	S	N	N	S	Position associated with antigenic drift, host specificity shift
T188A	T	T	T	A	T	T	A	Host specificity shift
N189D	N	N	N	D	N	N	D	Antigenic drift
220–224	NGQSG	NGQRG	NGQRG	NGQHG	NGQRG	NGQQ G	NGQRG	222–224 QS(R)G avian-like α2–3 receptor-binding preference
A263T	T	T	T	T	T	T	T	Increased virulence in mammals
Cleavage peptides	RERRRK R	REKRRK R	REKRRKR	RERRRKR	RERRRKR	RERRR KR	RERRRKR	Highly pathogenic avian influenza
NA (N6 no.)								
59–69 Del	yes	yes	no	yes	yes	yes	yes	Enhanced virulence in mice
N86K	N	K	N	K	N	K	K	Removes a potential N-glycosylation site
T223I	I	I	I	I	I	I	I	Increased virulence in mammals
PB2								
T63I	I	I	I	I	I	I	I	Increased virulence in mammals
L89V	V	V	V	V	V	V	V	Leu89Val, Gly309Asp, Thr339Lys, Arg477Gly, Ile495Val, Lys627Glu, Ala676Thr; enhanced polymerase activity and increased virulence in mice
G309D	D	D	D	D	D	D	D	Leu89Val, Gly309Asp, Thr339Lys, Arg477Gly, Ile495Val, Lys627Glu, Ala676Thr; enhanced polymerase activity and increased virulence in mice
T339K	K	K	M	K	T	K	K	Leu89Val, Gly309Asp, Thr339Lys, Arg477Gly,

Gene	Human H5N6 virus strains A/Hubei/ 29578/ 2016†	A/Fujian-Sanyuan/ 21099/ 2017†	A/Sichuan/ 26221/ 2014†	A/Guangxi/ 32797/ 2018†	Avian H5N6 virus strains A/chicken/ Vietnam/ NCVD-15A59/2015†	A/duck/ Hyogo/ 1/2016†	A/common gull/ Saratov/ 1676/2018	Phenotypic characteristics
								Ile495Val, Lys627Glu, Ala676Thr; enhanced polymerase activity and increased virulence in mice
Q368R	R	R	Q	R	Q	R	R	Increased virulence in mammals
H447Q	Q	Q	Q	Q	Q	Q	Q	Increased virulence in mammals
R477G	G	G	G	G	G	G	G	Leu89Val, Gly309Asp, Thr339Lys, Arg477Gly, Ile495Val, Lys627Glu, Ala676Thr; enhanced polymerase activity and increased virulence in mice
I495V	V	V	V	V	V	V	V	Leu89Val, Gly309Asp, Thr339Lys, Arg477Gly, Ile495Val, Lys627Glu, Ala676Thr; enhanced polymerase activity and increased virulence in mice
A/T588V	A	A	T	V	T	V	V	Host specificity shift
K627E	E	E	E	K	E	E	E	Enhanced polymerase activity and increased virulence in mice, adaptation to mammals
A661S	A	A	A	S	A	S	S	Host specificity shift
A676T	M	T	T	T	T	T	T	Leu89Val, Gly309Asp, Thr339Lys, Arg477Gly, Ile495Val, Lys627Glu, Ala676Thr; enhanced polymerase activity and increased virulence in mice
PB1								
A3V	V	V	V	V	V	V	V	Increased virulence in mammals
L13P	P	P	P	P	P	P	P	Increased virulence in mammals
R207K	K	K	K	K	K	K	K	Increased virulence in mammals
K328N	N	N	N	N	N	N	N	Increased virulence in mammals
I368V	V	I	I	I	I	I	I	Increased transmission in ferrets
S375N	N	N	N	N	N	N	N	Increased virulence in mammals
H436Y	Y	Y	Y	Y	Y	Y	Y	Increased virulence in mammals
L473V	V	V	V	V	V	V	V	Increased virulence in mammals
M677T	T	T	T	T	T	T	T	Increased virulence in mammals
PA								
V100A	I	V	V	V	V	V	V	Species associated signature position
G225S	S	S	G	S	S	S	S	Host specificity shift
H266R	R	R	R	R	R	R	R	Increased virulence in mammals
K356R	R	K	K	K	K	K	K	Species associated signature position
S409N	N	S	S	S	S	S	S	Species associated signature position
S/A515T	T	T	T	T	T	T	T	Increased virulence in mammals
NP								
I33V	V	V	I	V	V	V	V	Host specificity shift
M1								
V15I	I	V	I	I	I	I	I	Increased virulence in mammals
N30D	D	D	D	D	D	D	D	Increased virulence in mice
T215A	A	A	A	A	A	A	A	Increased virulence in mice
M2								
S31N	N	S	S	S	S	S	S	Resistance to adamantine
S89G	G	G	S	G	G	G	G	Host specificity shift
NSP1								
P42S	S	S	S	S	S	S	S	Increased virulence in mice
80–84 del	No	No	Yes	Yes	Yes	Yes	Yes	Increased virulence in mice
D92E‡	D	D	E	E	E	E	E	Increased virulence in mammals

Gene	Human H5N6 virus strains				Avian H5N6 virus strains			
	A/Hubei/ 29578/ 2016†	A/Fujian-Sanyuan/ 21099/ 2017†	A/Sichuan/ 26221/ 2014†	A/Guangxi/ 32797/ 2018†	A/chicken/ Vietnam/ NCVD-15A59/2015†	A/duck/ Hyogo/ 1/2016†	A/common gull/ Saratov/ 1676/2018	Phenotypic characteristics
L98F§	L	F	F	F	F	F	F	Increased virulence in mice
I101M§	I	M	M	M	M	M	M	Increased virulence in mice
V149A¶	A	A	A	A	A	A	A	Increased virulence in mammals
N200S§	S	S	S	S	S	S	S	Asn200Ser, when coupled with NS2 Thr47Ala; increased virulence in mammals
Terminal motif ESEV	Truncated	GSEV	ESEV	ESEV	ESEV	ESEV	ESEV	Increased virulence in mice
NSP2								
T47A	A	A	A	A	A	A	A	Thr47Ala (when coupled with NS1 Asn200Ser) Increased virulence in mammals

*Avian influenza A(H5N6) from this study, A/common gull/Saratov/1676/2018 in Global Initiative on Sharing All Influenza Data database. HA, hemagglutinin; M1, matrix 1; M2, matrix 2; NA, neuraminidase; NP, nucleoprotein; NSP1, nonstructural protein 1; NSP2, nonstructural protein 2;PA, polymerase; PB1, polymerase basic 1; PB2, polymerase basic 2.
†Candidate vaccine virus.
‡87 if the deletion is not counted.
§Deletion not counted.
¶144 if deletion not counted.

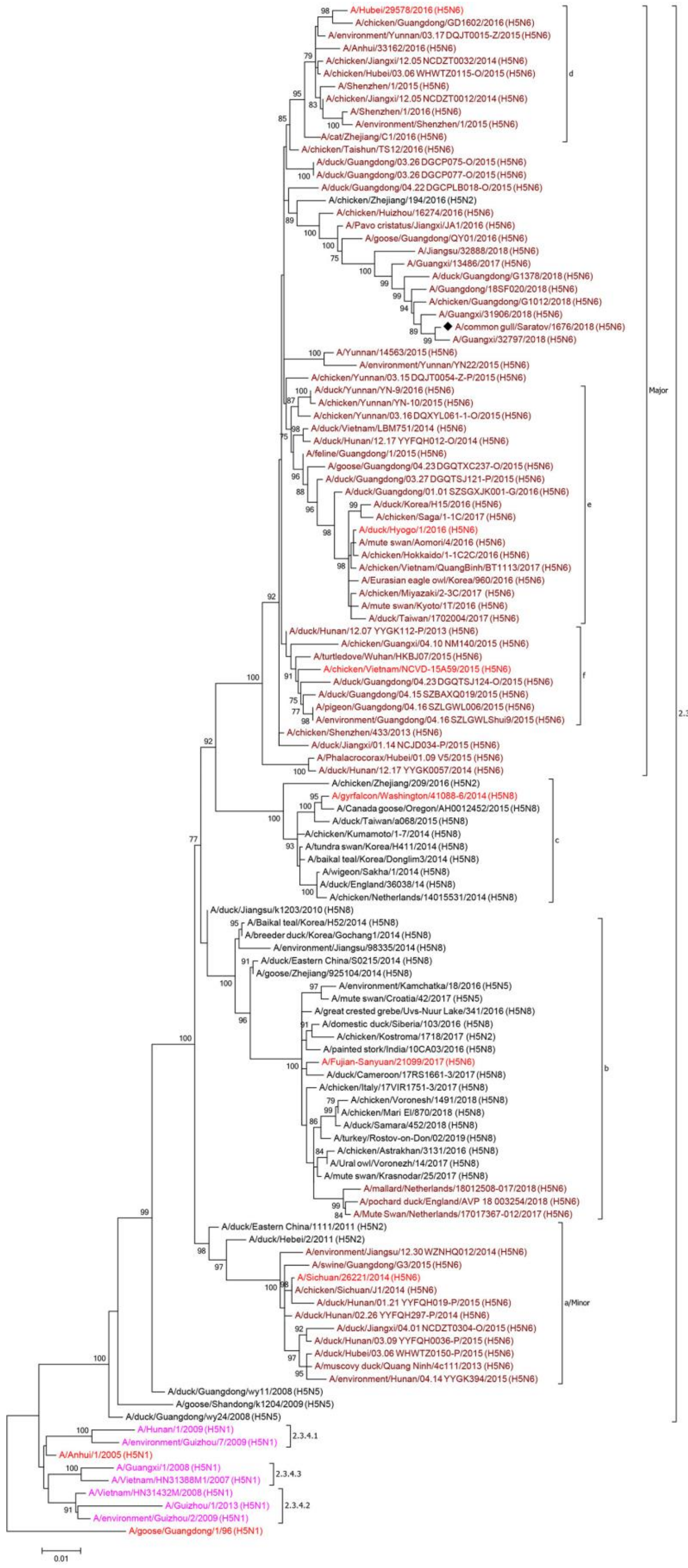

Appendix Figure 1. Phylogenetic analysis of the hemagglutinin (HA) gene segment of A/common gull/Saratov/1676/2018 (H5N6) isolated from a common gull (*Larus canus*) in the Saratov Region of Russia, 2018. Phylogenetic analysis was performed by using MEGA version 6.0 (http://www.megasoftware.net) and the maximum likelihood method with 500 bootstrap replications. Genetic clusters of avian influenza viruses are annotated by brackets. Numbers near the branches indicate bootstrap value >70%. Influenza virus sequences were deposited in Global Initiative on Sharing All Influenza Data (GISAID; https://platform.gisaid.org/epi3) under identification no. EPI1355418. Sequence data from the Influenza Research Database (IRD; https://www.fludb.org) and GenBank (https://www.ncbi.nlm.nih.gov/genbank) were used for comparison. Black diamond indicates isolate from this study. Red text indicates candidate vaccine viruses. Blue text indicates H9N2/H7N9 sequences; green text indicates H6 subtypes; pink text indicates H5N1 subtypes; brown text indicates H5N6 subtypes. Scale bar indicates nucleotide substitutions per site.

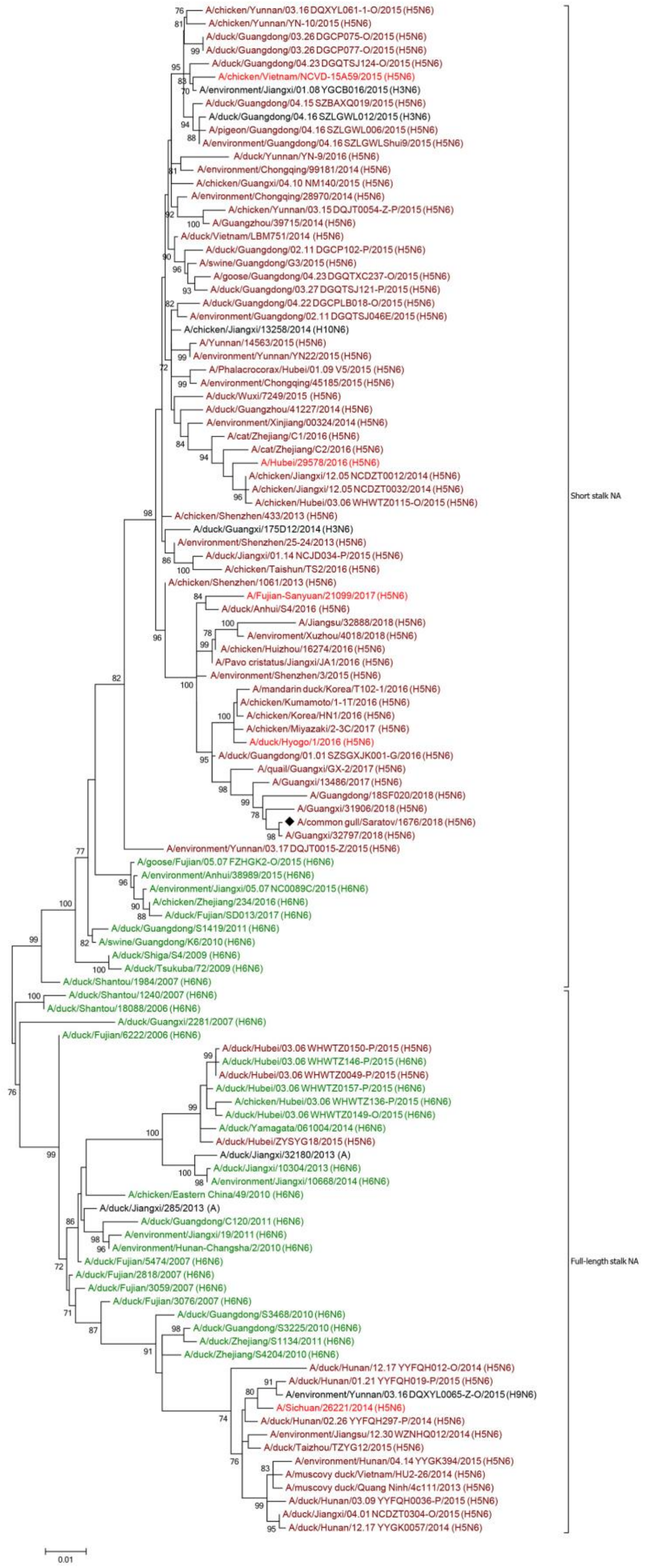

Appendix Figure 2. Phylogenetic analysis of the neuraminidase (NA) gene segment of A/common gull/Saratov/1676/2018 (H5N6) isolated from a common gull (*Larus canus*) in the Saratov Region of Russia, 2018. Phylogenetic analysis was performed by using MEGA version 6.0 (http://www.megasoftware.net) and the maximum likelihood method with 500 bootstrap replications. Genetic clusters of avian influenza viruses are annotated by brackets. Numbers near the branches indicate bootstrap value >70%. Influenza virus sequences were deposited in Global Initiative on Sharing All Influenza Data (GISAID; https://platform.gisaid.org/epi3) under identification no. EPI1355418. Sequence data from the Influenza Research Database (IRD; https://www.fludb.org) and GenBank (https://www.ncbi.nlm.nih.gov/genbank) were used for comparison. Black diamond indicates isolate from this study. Red text indicates candidate vaccine viruses. Blue text indicates H9N2/H7N9 sequences; green text indicates H6 subtypes; pink text indicates H5N1 subtypes; brown text indicates H5N6 subtypes. Scale bar indicates nucleotide substitutions per site.

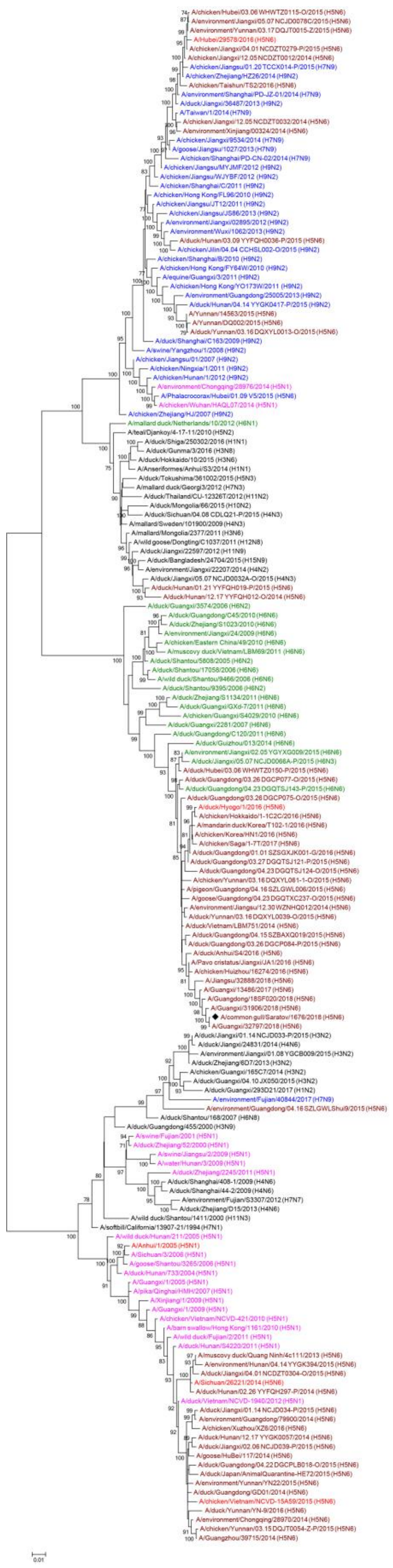

Appendix Figure 3. Phylogenetic analysis of the polymerase basic protein 2 (PB2) gene segment of A/common gull/Saratov/1676/2018 (H5N6) isolated from a common gull (*Larus canus*) in the Saratov Region of Russia, 2018. Phylogenetic analysis was performed by using MEGA version 6.0 (http://www.megasoftware.net) and the maximum likelihood method with 500 bootstrap replications. Numbers near the branches indicate bootstrap value >70%. Influenza virus sequences were deposited in Global Initiative on Sharing All Influenza Data (GISAID; https://platform.gisaid.org/epi3 under identification no. EPI1355418. Sequence data from the Influenza Research Database (IRD; https://www.fludb.org) and GenBank (https://www.ncbi.nlm.nih.gov/genbank) were used for comparison. Black diamond indicates isolate from this study. Red text indicates candidate vaccine viruses. Blue text indicates H9N2/H7N9 sequences; green text indicates H6 subtypes; pink text indicates H5N1 subtypes; brown text indicates H5N6 subtypes. Scale bar indicates nucleotide substitutions per site.

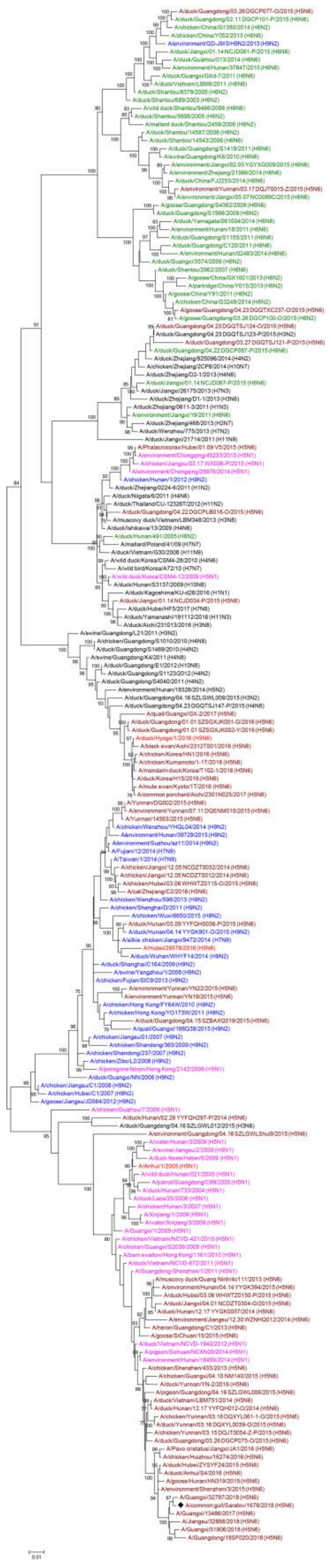

Appendix Figure 4. Phylogenetic analysis of the polymerase basic protein 1 (PB1) gene segment of A/common gull/Saratov/1676/2018 (H5N6) isolated from a common gull (*Larus canus*) in the Saratov Region of Russia, 2018. Phylogenetic analysis was performed by using MEGA version 6.0 (http://www.megasoftware.net) and the maximum likelihood method with 500 bootstrap replications. Numbers near the branches indicate bootstrap value >70%. Influenza virus sequences were deposited in Global Initiative on Sharing All Influenza Data (GISAID; https://platform.gisaid.org/epi3) under identification no. EPI1355418. Sequence data from the Influenza Research Database (IRD; https://www.fludb.org) and GenBank (https://www.ncbi.nlm.nih.gov/genbank) were used for comparison. Black diamond indicates isolate from this study. Red text indicates candidate vaccine viruses. Blue text indicates H9N2/H7N9 sequences; green text indicates H6 subtypes; pink text indicates H5N1 subtypes; brown text indicates H5N6 subtypes. Scale bar indicates nucleotide substitutions per site.

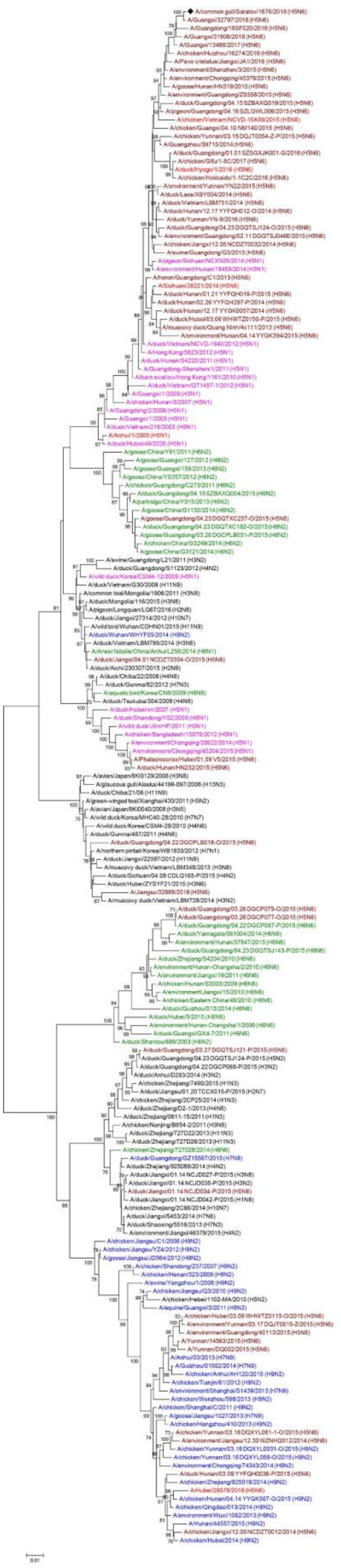

Appendix Figure 5. Phylogenetic analysis of the polymerase acidic (PA) gene segment of A/common gull/Saratov/1676/2018 (H5N6) isolated from a common gull (*Larus canus*) in the Saratov Region of Russia, 2018. Phylogenetic analysis was performed by using MEGA version 6.0 (http://www.megasoftware.net) and the maximum likelihood method with 500 bootstrap replications. Numbers near the branches indicate bootstrap value >70%. Influenza virus sequences were deposited in Global Initiative on Sharing All Influenza Data (GISAID; https://platform.gisaid.org/epi3) under identification no. EPI1355418. Sequence data from the Influenza Research Database (IRD; https://www.fludb.org) and GenBank (https://www.ncbi.nlm.nih.gov/genbank) were used for comparison. Black diamond indicates isolate from this study. Red text indicates candidate vaccine viruses. Blue text indicates H9N2/H7N9 sequences; green text indicates H6 subtypes; pink text indicates H5N1 subtypes; brown text indicates H5N6 subtypes. Scale bar indicates nucleotide substitutions per site.

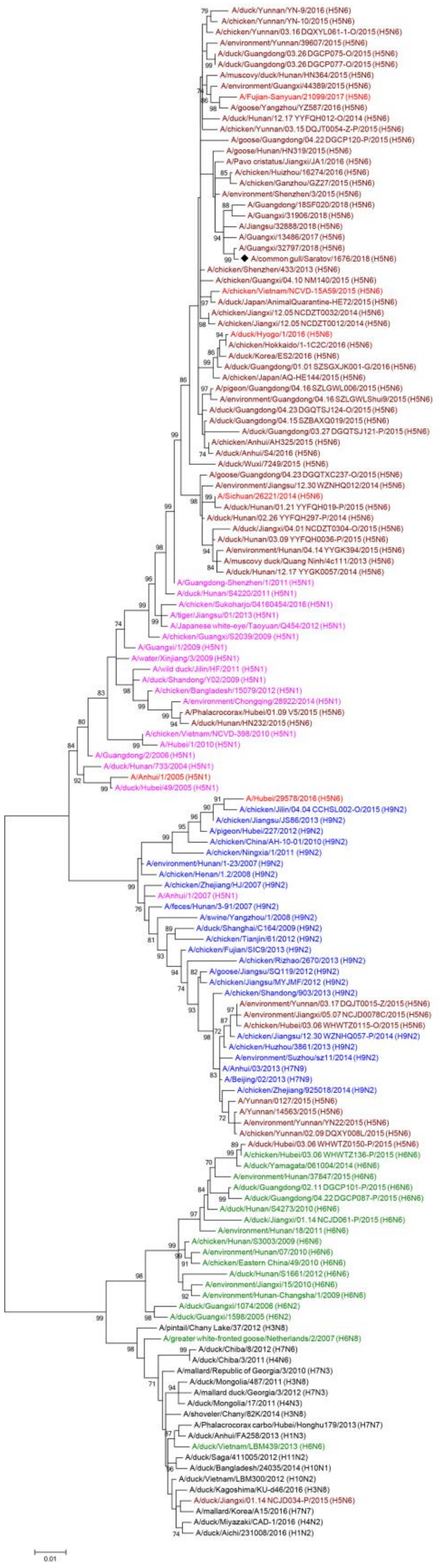

Appendix Figure 6. Phylogenetic analysis of the nucleoprotein (NP) gene segment of A/common gull/Saratov/1676/2018 (H5N6) isolated from a common gull (*Larus canus*) in the Saratov Region of Russia, 2018. Phylogenetic analysis was performed by using MEGA version 6.0 (http://www.megasoftware.net) and the maximum likelihood method with 500 bootstrap replications. Numbers near the branches indicate bootstrap value >70%. Influenza virus sequences were deposited in Global Initiative on Sharing All Influenza Data (GISAID; https://platform.gisaid.org/epi3) under identification no. EPI1355418. Sequence data from the Influenza Research Database (IRD; https://www.fludb.org) and GenBank (https://www.ncbi.nlm.nih.gov/genbank) were used for comparison. Black diamond indicates isolate from this study. Red text indicates candidate vaccine viruses. Blue text indicates H9N2/H7N9 sequences; green text indicates H6 subtypes; pink text indicates H5N1 subtypes; brown text indicates H5N6 subtypes. Scale bar indicates nucleotide substitutions per site.

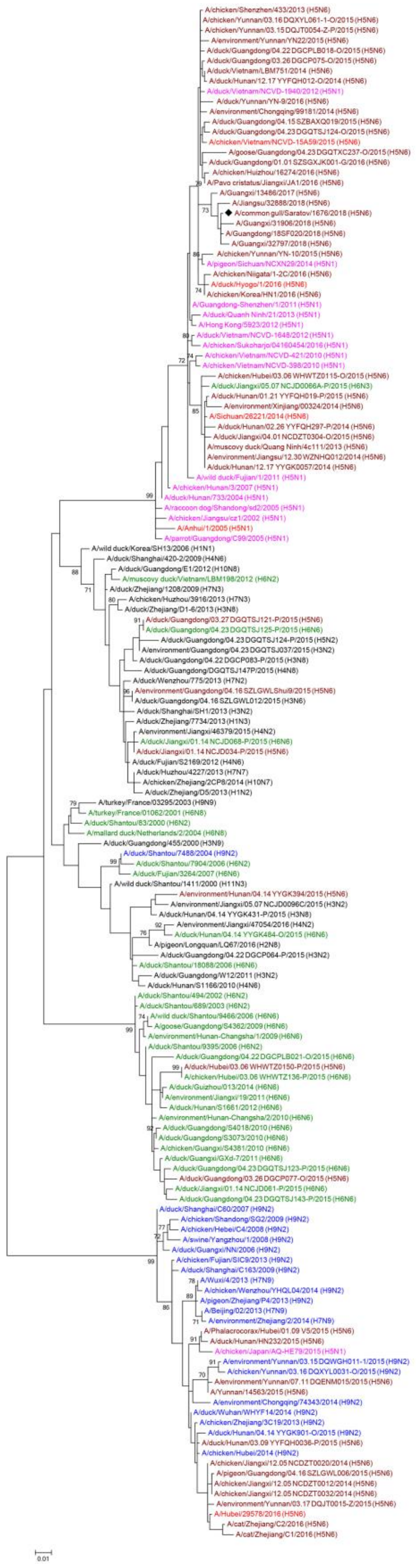

Appendix Figure 7. Phylogenetic analysis of the matrix (M) gene segment of A/common gull/Saratov/1676/2018 (H5N6) isolated from a common gull (*Larus canus*) in the Saratov Region of Russia, 2018. Phylogenetic analysis was performed by using MEGA version 6.0 (http://www.megasoftware.net) and the maximum likelihood method with 500 bootstrap replications. Numbers near the branches indicate bootstrap value >70%. Influenza virus sequences were deposited in Global Initiative on Sharing All Influenza Data (GISAID; https://platform.gisaid.org/epi3) under identification no. EPI1355418. Sequence data from the Influenza Research Database (IRD; https://www.fludb.org) and GenBank (https://www.ncbi.nlm.nih.gov/genbank) were used for comparison. Black diamond indicates isolate from this study. Red text indicates candidate vaccine viruses. Blue text indicates H9N2/H7N9 sequences; green text indicates H6 subtypes; pink text indicates H5N1 subtypes; brown text indicates H5N6 subtypes. Scale bar indicates nucleotide substitutions per site.

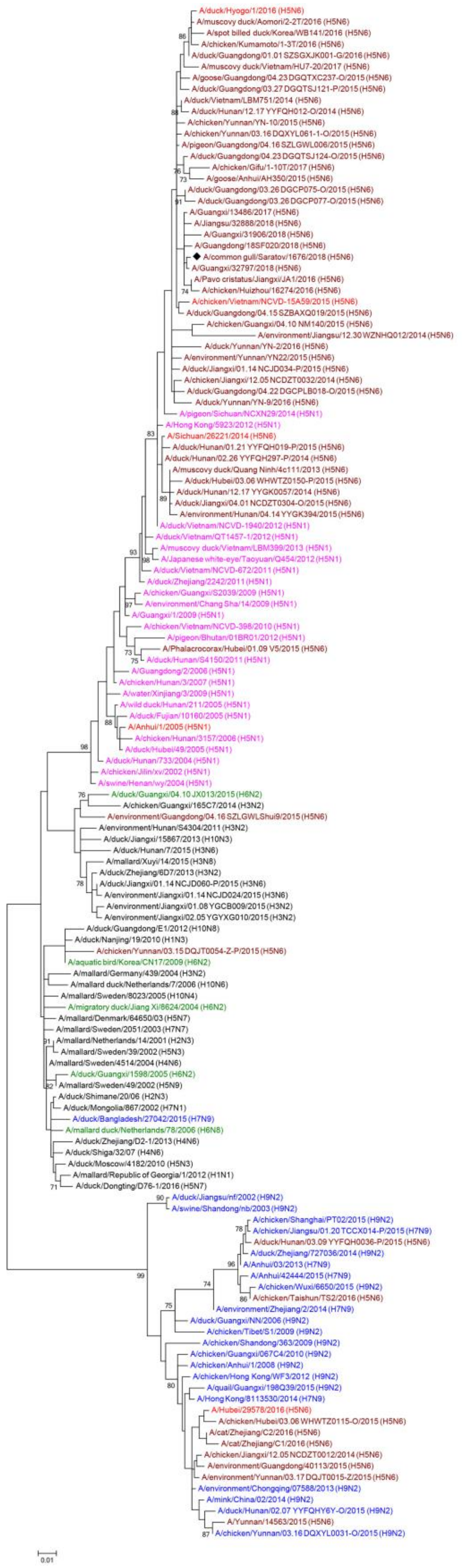

Appendix Figure 8. Phylogenetic analysis of the nonstructural protein (NSP) gene segment of A/common gull/Saratov/1676/2018 (H5N6) isolated from a common gull (*Larus canus*) in the Saratov Region of Russia, 2018. Phylogenetic analysis was performed by using MEGA version 6.0 (http://www.megasoftware.net) and the maximum likelihood method with 500 bootstrap replications. Numbers near the branches indicate bootstrap value >70%. Influenza virus sequences were deposited in Global Initiative on Sharing All Influenza Data (GISAID; https://platform.gisaid.org/epi3) under identification no. EPI1355418. Sequence data from the Influenza Research Database (IRD; https://www.fludb.org) and GenBank (https://www.ncbi.nlm.nih.gov/genbank) were used for comparison. Black diamond indicates isolate from this study. Red text indicates candidate vaccine viruses. Blue text indicates H9N2/H7N9 sequences; green text indicates H6 subtypes; pink text indicates H5N1 subtypes; brown text indicates H5N6 subtypes. Scale bar indicates nucleotide substitutions per site.

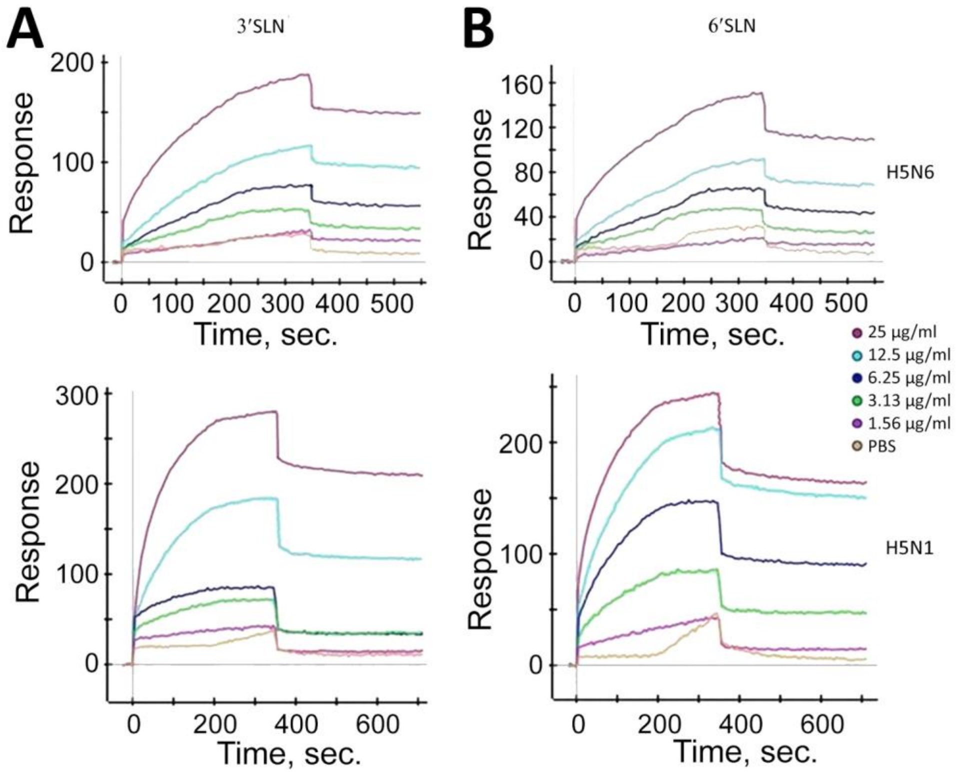

Appendix Figure 9. Surface plasma resonance sensorgrams for interaction of A/common gull/Saratov/1676/2018 (H5N6) and A/rook/Chany/32/2015 (H5N1) using receptor analogs for A) 3′SLN and B) 6′SLN after injection of viruses at the indicated concentrations. We used phosphate-buffered saline as a reference, which indicated specific binding between the virus and glycans. PBS, phosphate-buffered saline.
